# Development of animal models to study aggressive thyroid cancers

**DOI:** 10.1530/ETJ-24-0361

**Published:** 2025-02-10

**Authors:** Shovan Dutta, Jeffrey A Knauf

**Affiliations:** ^1^ Center for Immunotherapy and Precision Immuno-Oncology, Cleveland Clinic, Cleveland, Ohio, USA; ^2^ Lerner Research Institute, Cleveland Clinic, Cleveland, Ohio, USA; ^3^ Department of Otolaryngology-Head & Neck Surgery, Cleveland Clinic Lerner College of Medicine of Case Western Reserve University, Cleveland, Ohio, USA

**Keywords:** thyroid cancer, BRAF, RAS, mouse models, anaplastic thyroid cancer

## Abstract

The development of mouse models for thyroid cancer has significantly advanced over the years, enhancing our understanding of thyroid tumorigenesis, molecular pathways and treatment responses. The earliest mouse models of thyroid cancer relied on hormone, radiation or chemical carcinogenesis to induce tumors. However, as our understanding of the genetic alterations driving thyroid cancer has expanded, more sophisticated genetic engineering techniques have been employed to create models with thyroid-specific expression of these driver mutations. While driver mutations can initiate tumorigenesis, they are often insufficient to sustain cancer progression and invasion, which significantly limits their usefulness in studying advanced thyroid cancers. Recent studies exploring the genomic landscape of advanced thyroid cancer have identified several cooperating mutations, which are secondary genetic alterations that work alongside driver mutations to promote thyroid tumor progression. Indeed, mice with a combination of oncogenic drivers and common cooperating alterations have been developed, demonstrating that these alterations function in conjunction with the oncogenic driver to promote the progression to advanced thyroid cancer. These models provide important preclinical tools to explore how cooperating alterations influence the response to therapies, particularly those targeting the oncogenic driver. This review will focus on recent publications that broaden the scope of advanced thyroid cancer models by combining thyroid-specific oncogenic driver expression with various cooperating mutations.

## Introduction

Over the past three decades, there has been a marked improvement in animal models of thyroid cancer. The earliest mouse models relied on hormone, radiation or chemical carcinogenesis to induce thyroid tumors. These models lacked specificity for human thyroid cancer mutations and primarily focused on understanding tumor initiation and progression (1). Their limitations included high variability, long latency, poor penetrance and in most cases, limited relevance to the genetic basis of human thyroid cancer. However, they laid the groundwork for the study of thyroid tumorigenesis. Following this, simple transgenic models were developed using promoters of thyroid-specific genes (e.g. thyroglobulin) to overexpress oncogenes identified in early genetic screens (e.g. HRAS^G12V^, RET gene rearrangements, and BRAF^V600E^), specifically in mouse thyroid follicular cells. These models provided valuable insight into roles of these oncogenes in thyroid tumorigenesis. However, the oncogenes were typically overexpressed and not under the control of their endogenous promoters. In addition, many of the oncogenes induced thyroid dedifferentiation, which in turn decreased their expression. These early models (Table 1) have been extensively reviewed in previous articles and will not be discussed in detail here (2, 3, 4, 5).

**Table 1:** Mouse models of thyroid cancer mediated by the expression of oncogenic driver guided by studies on the genomic landscape of human thyroid cancer.

Mouse model	Description	Year	Reference
*Tg-RET-PTC1*	Human RET/PTC1 fusion under control of the bovine thyroglobulin promoter. Mice develop PTCs	1996	(95, 96)
*TG-HRAS* ^*G12V*^	Human HRAS^G12V^ under control of the bovine thyroglobulin promoter. Mice develop PTCs	1996	(97)
*TG-RET-PTC3*	Human RET/PTC3 fusion under control of the bovine thyroglobulin promoter. Mice develop PTCs	1998	(98)
*TG-TRK-T1*	The human TRK-T1 oncogene, a fusion of TPR with NTRK1, under control of the bovine thyroglobulin. Mice develop PTCs	2000	(99)
*TG-BRAF* ^*V600E*^	Human BRAF^V600E^ under control of the bovine thyroglobulin promoter. Mice develop PTCs that progress to PDTCs	2005	(100, 101)
*TG-Nras* ^*G61L*^	Human NRAS^G61L^ under control of the bovine thyroglobulin promoter. Mice develop FTCs that progress to PDTCs	2006	(102)
*TPO-Cre/LSL-Kras* ^*G12D*^	Mice with thyroid-specific knock-in of oncogenic *Kras*^*G12D*^. Oncogenic Kras unable to transform thyroid follicular cells when expressed at endogenous levels	2009	(50)
*TPO-Cre/LSL-Braf*	Mice with thyroid-specific knock-in of oncogenic *Braf*^*V600E*^. Mice develop early onset PTC with near 100% penetrance	2011	(30)
*TG-CreER* ^*T2*^ */Braf* ^*CA*^	Mice with thyroid-specific knock-in of oncogenic *Braf*^*V600E*^. Mice develop late onset PTC with near 100% penetrance	2011	(103)
*TPO-Cre/FR-Hras* ^*G12V*^	Mice with thyroid-specific knock-in of oncogenic *Hras*^*G12V*^. Oncogenic Hras unable to transform thyroid follicular cells when expressed at endogenous levels	2011	(30)
*Tg-RTTA/tetO-mycBRAF* ^*V600E*^	Thyroid-specific inducible expression of human BRAF^V600E^	2011	(104)
*TPO-CreER* ^*T2*^ */Braf* ^*CA*^	Mice with thyroid-specific knock-in of oncogenic *Braf*^*V600E*^. Mice develop late onset PTC with near 100% penetrance	2014	(33)
*Tg-STRN-ALK*	Human STRN/ALK fusion under control of the bovine thyroglobulin promoter. Mice develop PDTC after ∼12 months	2018	(105)
*Nkx2*.*1-CreER*^*T2*^*/Braf*^*CA*^	Mice with lung and thyroid knock-in of oncogenic *Braf*^*V600E*^. Mice develop multifocal PTC and lung adenocarcinomas with near 100% penetrance	2023	(106)

PTC, papillary thyroid carcinoma.

**Figure 1:**
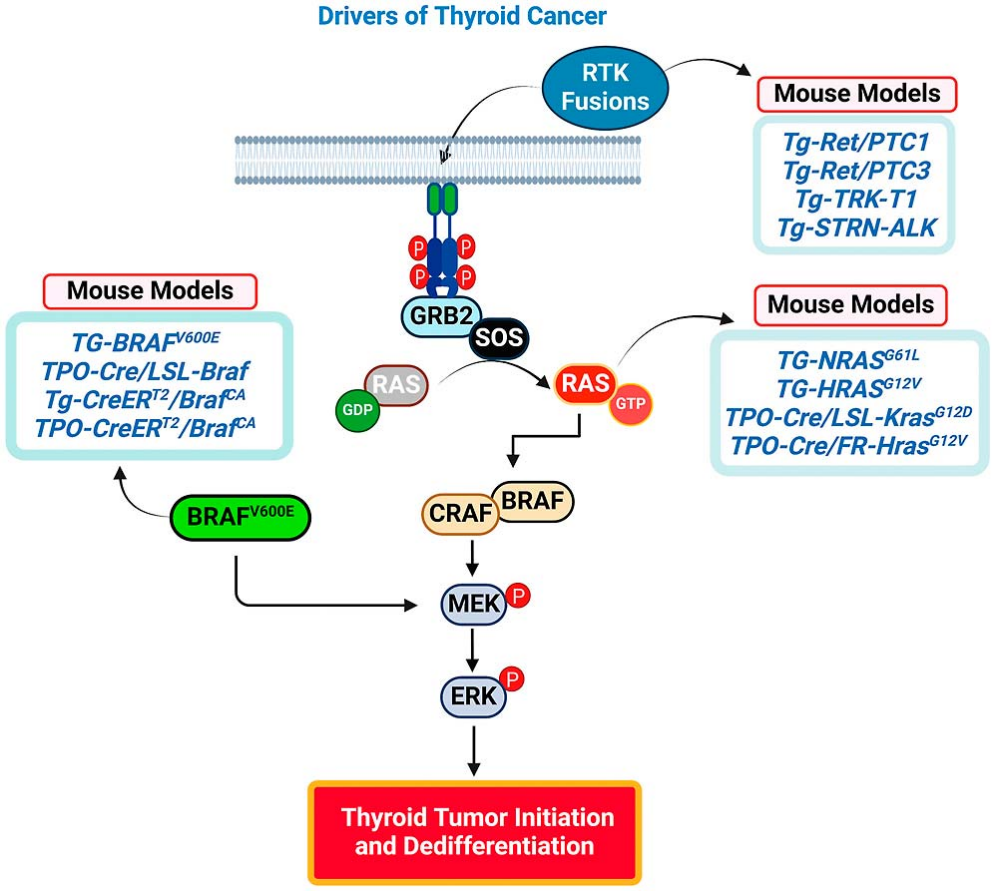
Mouse models of thyroid cancer oncogenic drivers. These models serve as an essential starting point for developing the most accurate mouse models of advanced thyroid cancer within the context of a fully functional immune system. The illustration was created using BioRender.com.

With advances in genetic engineering, more accurate models mimicking human thyroid cancers have emerged. These include transgenic mice with knock-in mutations of oncogenic drivers such as BRAF^V600E^, HRAS^G12V^, and KRAS^G12D^, which are the most common oncogenic drivers in human thyroid cancers (6, 7, 8, 9). These models (Table 1), which almost universally activate the MAPK pathway (Fig. 1), have enabled the study of driver mutations, tumor microenvironments, metastasis and mechanism of therapy resistance with the genes expressed under the control of their endogenous promoters (reviewed in (5, 10, 11, 12, 13)). However, while driver mutations can initiate tumorigenesis, they are often insufficient to sustain cancer progression and invasion. This is where cooperating mutations become crucial. Cooperating mutations are secondary genetic alterations that work alongside driver mutations to increase malignancy. These mutations typically do not independently initiate cancer, but when they are present in combination with driver mutations, they can exacerbate tumor aggressiveness, enhance metastatic potential and contribute to treatment resistance.

**Figure 2:**
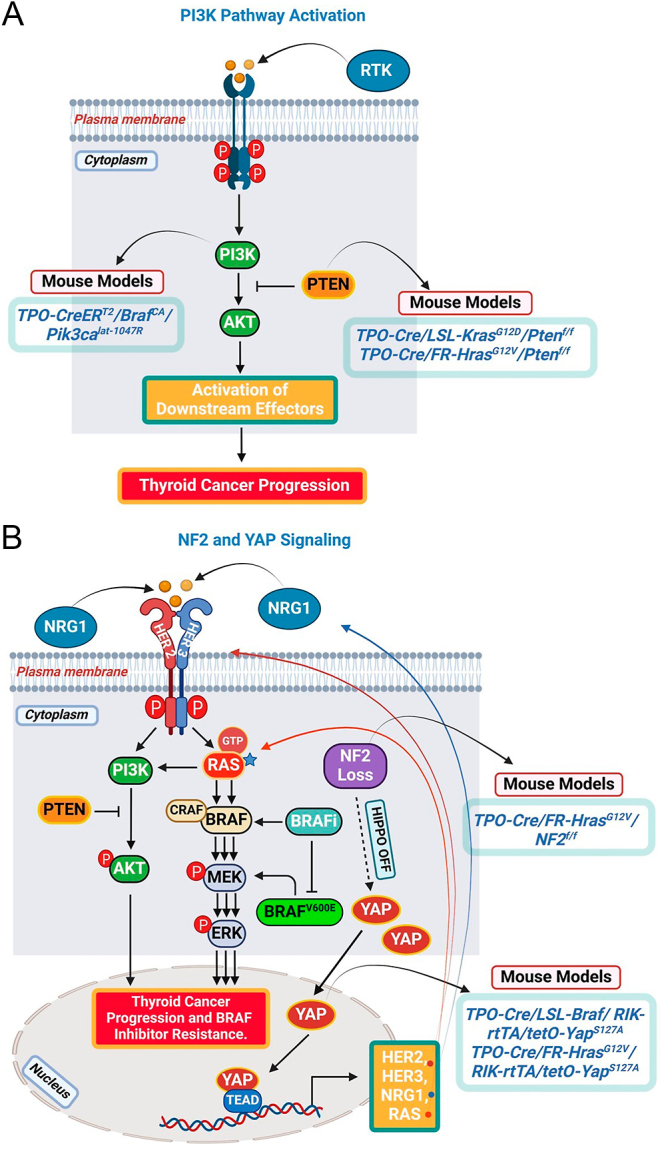
Mouse models of advanced thyroid cancer generated by combining oncogenic drivers with pathway-activating cooperating alterations. (A) PI3K pathway activation via PIK3CA activating mutations or PTEN loss. (B) YAP activation through NF2 loss or a YAP mutation that promotes its nuclear localization, increasing the expression of transcriptional targets such as HER2, HER3, NRG1 and RAS. These, in turn, promote resistance to BRAF inhibitors in BRAF^V600E^-driven thyroid cancer. In the context of mutant RAS (*), YAP activation drives the overexpression of oncogene, promoting thyroid cancer progression. The illustration was created using BioRender.com.

**Figure 3:**
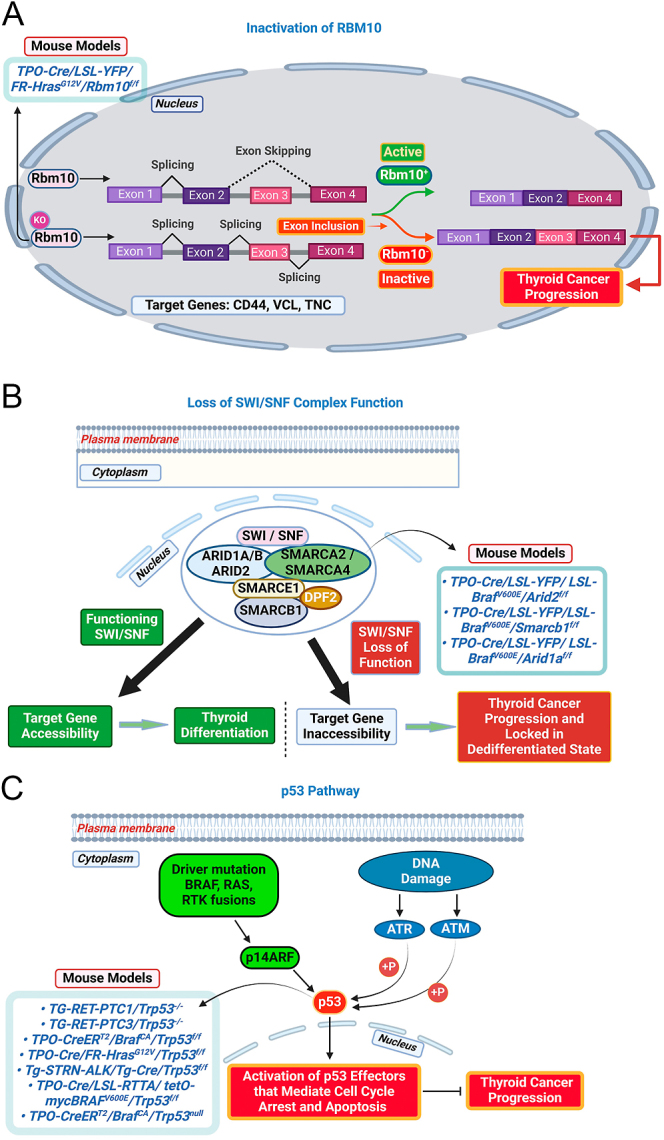
Mouse models of advanced thyroid cancer generated by combining oncogenic drivers with pathway-inactivating cooperating alterations. (A) RBM10 loss-of-function. RBM10 regulates mRNA splicing of target genes, and its dysfunction leads to alternative splicing, primarily through exon inclusion. The alternatively spliced gene products exhibit altered activity, which cooperates to promote thyroid tumor progression. (B) SWI/SNF complex loss-of-function. The SWI/SNF complex, consisting of 12–15 subunits, mediates nucleosome mobilization and chromatin remodeling. Alterations in key subunits disrupt complex function, and when combined with BRAF^V600E^, they promote tumor progression. In addition, loss of SWI/SNF function alters chromatin structure, locking thyroid cells in a dedifferentiated state. (C) Loss of p53 function. p53 regulates cell cycle arrest and apoptosis in response to cellular stress, such as DNA damage or oncogene activation. Loss of p53 function promotes genomic instability and diminishes oncogene-induced senescence, which cooperates with oncogenic drivers to accelerate thyroid tumor progression. The illustration was created using BioRender.com.

The arrival of advanced genomic sequencing technologies has significantly expanded our understanding of the alterations that cooperate with driver mutations, playing essential roles in thyroid cancer progression and in some cases, contribute to the development of deadly anaplastic thyroid carcinomas (14, 15, 16, 17, 18, 19, 20, 21). These alterations include TERT promoter mutations, loss-of-function mutations in tumor suppressor genes such as TP53, PTEN and RBM10, gain of function mutations in PI3K pathway genes and EIF1AX and mutations in genes involved in epigenetic regulation (7, 8, 9, 22, 23, 24, 25). The initial generation of these combined transgenic mice (reviewed in (10, 21, 26, 27)), which included oncogenic *Braf*, *Kras* and *Hras* knock-ins, with the common cooperating alterations found in human genomic studies of advanced thyroid cancer (i.e. TERT promoter, TP53, PI3K pathway genes and EIF1AX), confirmed the ability of these cooperating mutations to promote tumor progression when combined with driver alterations. These models have also provided critical insights into the response to therapies targeting the oncogenic driver (Table 2, Figs 2 and 3). This review will focus on recent studies that have utilized findings from genomic landscape analysis of advanced thyroid cancer to guide the expanded development of mouse models for advanced thyroid cancers. These models integrate knock-in mutations of thyroid cancer drivers with additional alterations suggested by genomic studies of advanced thyroid cancer, aiming to better recapitulate thyroid cancer progression.

**Table 2:** Mouse models of advanced thyroid cancers mediated by the expression of oncogenic driver in combination with cooperating alterations identified by genomic landscape studies of advanced human thyroid cancer.

Mouse model	Description	Year	Reference
*TG-RET-PTC1/Trp53^−^* ^*/*^ *^−^*	Human RET/PTC1 fusion under control of the bovine thyroglobulin promoter and global knockout of *Trp53*. Mice develop PTCs that progress to ATCs	2000	(107)
*TG-RET-PTC3/Trp53^−^* ^*/*^ *^−^*	Human RET/PTC3 fusion under control of the bovine thyroglobulin promoter and global knockout of *Trp53*. Mice develop PTCs that progress to PDTCs	2001	(108)
*TPO-Cre/LSL-Kras* ^*G12D*^ */Pten* ^*f/f*^	Mice with thyroid-specific knock-in of oncogenic *Kras*^*G12D*^ and loss of *Pten*. Mice develop FTCs that progress to PDTCs.	2009	(50)
*TPO-CreER* ^*T2*^ */Braf* ^*CA*^ */Trp53* ^*f/f*^	Thyroid-specific knock-in of oncogenic *Braf*^*V600E*^ and loss *Trp53*. Mice develop PTCs with near 100% penetrance that progress to ATCs	2014	(33)
*TPO-CreER* ^*T2*^ */Braf* ^*CA*^ */Pten* ^*f/f*^	Conditional thyroid-specific knock-in of *Braf*^*V600E*^ and loss of *Pten*. Mice develop PTCs that progress to ATCs	2014	(109)
*TPO-CreER* ^*T2*^ */Braf* ^*CA*^ */Pik3ca* ^*lat*^ *^−1047^* ^*R*^	Conditional thyroid-specific knock-in of both *Braf*^*V600E*^ and *Pik3ca*^*H1047R*^. Mice develop PTCs that progress to ATCs	2014	(109)
*TPO-Cre/FR-Hras* ^*G12V*^ */Nf2* ^*f/f*^	Thyroid-specific knock-in oncogenic *Hras*^*G12V*^ and loss of *Nf2*. Mice develop poorly differentiated thyroid cancer with a high penetrance	2015	(52)
*TPO-Cre/FR-Hras* ^*G12V*^ */Pten* ^*f/f*^	Thyroid-specific knock-in oncogenic *Hras*^*G12V*^ and loss of *Pten*. Mice develop poorly differentiated thyroid cancer with a high penetrance. Mice develop FTC that metastasize	2015	(52, 110)
*TPO-CreER* ^*T2*^ */Braf* ^*CA*^ */Trp53* ^*null*^	Thyroid-specific knock-in of oncogenic *Braf*^*V600*E^ and global knockout of *Trp53*. Mice develop PTCs with near 100% penetrance that progress to ATCs	2016	(34)
*TPO-Cre/LSL-RTTA/tetO-mycBRAF* ^*V600E*^ */Trp53* ^*f/f*^	Thyroid-specific inducible expression human oncogenic BRAF^V600E^ and loss of p53. Mice develop *de novo* ATCs (PTCs not precursor) with a 8–10 weeks latency	2018	(111)
*TPO-Cre/FR-Hras* ^*G12V*^ */Trp53* ^*f/f*^	Thyroid-specific knock-in oncogenic *Hras*^*G12V*^ and loss of *Trp53*. Mice develop PDTCs and ATCs	2018	(112)
*Tg-STRN-ALK/Tg-Cre/Trp53* ^*f/f*^	Human STRN/ALK fusion under control of the bovine thyroglobulin promoter and thyroid-specific loss of Trp53. Mice develop PDTC after ∼6 months	2019	(113)
*TPO-Cre/LSL-YFP/LSL-Braf* ^*V600E*^ */Arid2* ^*f/f*^	Thyroid-specific knock-in of *Braf*^*V600E*^ and loss of the SWI/SNF subunit *Arid2*. Mice develop early onset PTC that progress to PDTC that are refractory to the restoration of iodide uptake by MAPK pathway inhibitors	2021	(114)
*TPO-Cre/LSL-YFP/LSL-Braf* ^*V600E*^ */Smarcb1* ^*f/f*^	Thyroid-specific knock-in of *Braf*^*V600E*^ and loss of the SWI/SNF subunit *Smarcb1*. Mice develop early onset PTCs that progress to PDTCs and ATCs that are refractory to the restoration of iodide uptake by MAPK pathway inhibitors	2021	(114)
*TPO-Cre/LSL-YFP/LSL-Braf* ^*V600E*^ */Arid1a* ^*f/f*^	Thyroid-specific knock in of *Braf*^*V600E*^ and loss of the SWI/SNF subunit *Arid1a*. Mice develop early onset PTCs that progress to PDTCs that are refractory to the restoration of iodide uptake by MAPK pathway inhibitors	2021	(114)
*TPO-Cre/LSL-Braf/RIK-rtTA/tetO-Yap* ^*S127A*^	Thyroid-specific knock-in of oncogenic *Braf*^*V600E*^ and inducible expression of constitutively active YAP^Y127A^. Mice develop PTCs with near 100% penetrance that progress to ATCs with metastasis	2022	(72)
*TPO-Cre/FR-Hras* ^*G12V*^ */RIK-rtTA/tetO-Yap* ^*S127A*^	Thyroid-specific knock-in of oncogenic *Hras*^*G12V*^ and inducible expression of constitutively active Yap^Y127A^. Mice develop PDTCs that progress to ATCs with metastasis	2022	(72)
*Braf^CA^*/Mieap^KO^	Global knockout of Mieap and thyroid-specific knock-in of *Braf*^*V600E*^ mediate by injecting Ad–TgP–Cre (adenovirus express Cre under control of thyroglobulin) into thyroid	2022	(76)
*Braf* ^*CA*^ */Atg5* ^*f/* *f*^	Thyroid-specific knock-in of BRAF^V600E^ and loss of Atg5 mediate by injecting Ad–TgP–Cre (adenovirus express Cre under control of thyroglobulin) into thyroid	2022	(76)
*TPO-Cre/LSL-YFP/LSL-Braf* ^*V600E*^ */Tert* ^*123C>T*^	Thyroid-specific knock-in of oncogenic Braf^V600E^ and global Tert promoter mutation. Mice develop early onset PTCs with near 100% penetrance with progression to PDTCs and a subset progressing to ATC	2023	(115)
*TPO-Cre/LSL-YFP/LSL-Braf* ^*V600E*^ */K5-Tert*	Thyroid-specific knock-in of oncogenic Braf^V600E^ and Tert overexpression epithelial tissue via the keratin 5 promoter. Mice develop early onset PTCs with near 100% penetrance with progression to PDTCs and a subset progressing to ATC	2023	(115)
*TPO-Cre/LSL-YFP/FR-Hras* ^*G12V*^ */Rbm10* ^*f/f*^	Thyroid-specific knock-in oncogenic Hras^G12V^ and loss of Rbm10. Mice develop PTCs with majority progressing to ATC with metastasis	2024	(55)
*TPO-Cre/LSL-Braf* ^*V600E*^ */Mettl3* ^*f/f*^	Thyroid-specific knock-in of oncogenic Braf^V600E^ and loss of Mettl3. Mice develop early onset PTCs with near 100% penetrance. Loss of Mettl3 increases tumor growth and shortens survival	2024	(64)
*TPO-Cre/LSL-Braf* ^*V600E*^ */Ng2* ^*f/f*^	Thyroid-specific knock-in of oncogenic Braf^V600E^ and loss of Ng2. Loss of Ng2 increases the response to Braf inhibitor by blocking feed-back induced RTK activation	2024	(71)

PTC, papillary thyroid carcinoma; PDTC, poorly differentiated thyroid cancer; RTK, receptor tyrosine kinase; ATG5, autophagy-related gene 5; ATC, anaplastic thyroid cancer.

## Building mouse models of advanced thyroid cancer

The MAPK pathway plays a pivotal role in the initiation, progression and therapeutic response of thyroid cancers, particularly follicular thyroid cancer (FTC), papillary thyroid cancer (PTC), and anaplastic thyroid cancer (ATC). The identification of driver mutations that activate this pathway, such as BRAF, RAS mutations and RET/PTC rearrangements, has provided critical insights into thyroid cancer biology and facilitated the development of targeted therapies. Consequently, models that mimic these oncogenic events form the foundation for generating mouse models of advanced thyroid cancer. Mouse models with the MAPK pathway drivers under the control of a thyroid-specific promoter (i.e. thyroglobulin) have been developed. However, in these models, the expression of the oncogene can be reduced or lost as the thyroid tumor progresses due to loss of thyroid differentiation, complicating interpretation of results. For this reason, thyroid-specific oncogene knock-in mouse models where the oncoprotein is regulated by its endogenous promoter are considered as optimal starting points for generating mouse models of advanced thyroid cancers. To date, knock-in models of the most common oncogenic drivers in thyroid cancer have been used to study thyroid cancer and these include *Braf*^*V600E*^, *Hras*^*G12V*^ and *Kras*^*G12D*^.

### Oncogenic Braf knock-in models: the foundation for models of BRAF-driven advanced thyroid cancers

The first conditional knock-in of *Braf*^*V600E*^ (*LSL-Braf*) was developed by Mercer *et al.* (28). In this line, exon 15 of the mouse genome was replaced with a floxed cDNA cassette encoding exons 15–18 of the wild-type mouse Braf, followed by a mouse genomic fragment of exon 15 containing the T1799A mutation, which encodes for BRAF^V600E^. A second line, *Braf*^*CA*^, developed by Dankort *et al.* (29), had a nearly identical design. Initial reports using these two lines to generate mice thyroid-specific knock-in of *Braf*^*V600E*^ reported a marked difference in the time to PTC development. *TPO-Cre/LSL-Braf* mice showed nearly 100% of the animals that developed PTC at five weeks (30). In contrast, 50% of *Tg-CreER*^*T2*^*/Braf*^*CA*^ mice developed PTC only six months after the Cre-mediated expression of Braf^V600E^. This discrepancy is attributed to the different transgenes used to drive Cre recombinase expression in thyroid follicular cells. In *TPO-Cre/LSL-Braf* mice, Cre recombinase is under the control of thyroid peroxidase promoter, which drives Cre expression and induction of BRAF^V600E ^starting around embryonic day 14.5 (31). In contrast, Dankort *et al.* (29) used a tamoxifen-inducible Cre under the control the thyroglobulin promoter, resulting in BRAF^V600E^ expression in thyroid follicular cells when the mice were 6–8-weeks-old. Indeed, a subsequent study showed that PTC development occurred around eight months after treating mice generated by crossing *TPO-CreER*^*T2*^ with *LSL-Braf* with tamoxifen compared to just five weeks in mice generated by crossing *TPO-Cre* with *LSL-Braf* (32). The reason behind the marked difference in latency of PTC development, influenced by the timing of Cre expression, remains unclear.

One notable difference between the lines is that the exon 15–24 cDNA fragment in the *LSL-Braf* mice does not function properly, resulting in a null *Braf* allele before recombination. The impact of this on BRAF^V600E^-induced thyroid tumorigenesis or therapeutic responses, if any, is unknown. However, as mice homozygous for *LSL-Braf* are not viable, it can pose challenges when creating multitransgenic mouse models that combine the Braf^V600E^ knock-in with other genetic alterations to study advanced thyroid cancer.

Both *Braf*^*CA*^ and *LSL-Braf* appear equally suitable for developing mouse models of BRAF^V600E^-driven advanced thyroid cancers. When crossed with *p53-flox* or *p53* knockout mice, the loss of p53 synergizes with the BRAF^V600E^ creating a more aggressive PTC that progresses to poorly differentiated thyroid cancer (PDTC) and eventually ATCs (33, 34, 35). The latency for ATC development was significantly shorter in *TPO-Cre/LSL-Braf/p53*^*−/−*^ mice, with ∼50% of the mice developing ATC at 18 weeks (34), compared to *TPO-CreER*^*T2*^*/Braf*^*CA*^*/p53*^*f/f*^, where ∼50% of the mice developed ATC at 24 weeks (33). This was likely due to the timing of *Braf*^*V600E*^ knock-in, as the conditional *TPO-CreER*^*T2*^ was used to promote follicular cell expression of BRAF^V600E^ at 6–8 weeks in the case of *Braf*^*CA*^. Whereas, *TPO-Cre* used in the *LSL-Braf* mice inducing follicular cell BRAF^V600E^ expression starting around embryonic day 14.5 (31). Indeed, when *Braf*^*CA*^ mice were crossed to generate *TPO-Cre/Braf*^*CA*^*/p53*^*f/f*^ mice (Knauf, unpublished observations), ATC development began as early as ∼12 weeks, with 50% of mice developing ATCs by 18 weeks of age, similar to what is observed with *TPO-Cre/LSL-Braf/p53*^*−/−*^ mice. While the kinetics differ slightly among the models, they all initially develop PTC, which subsequently progresses to ATC, consistent with the stepwise progression seen in human thyroid cancer. Immunohistochemistry staining for F4/80 and CD206 by Yan *et al.* (35) in ATCs from *TPO-Cre/LSL-Braf/p53*^*−/−*^ mice, along with immune deconvolution of RNAseq data from ATCs in *TPO-CreER*^*T2*^*/Braf*^*CA*^*/p53*^*f/f*^ mice (27), revealed high tumor infiltration of M2-like macrophages, similar to what has been observed in human ATCs (36, 37, 38).

In addition to *p53-flox* mice, the Braf knock-in lines (either *Braf*^*CA*^ or *LSL-Braf*) have been crossed with other cooperating alterations to generate multitransgenic models, promoting more aggressive PTC that progresses to either PDTC or ATC. This underscores the utility of *Braf*^*V600E*^ knock-in mice as a key starting point for developing advanced thyroid cancer models to study advanced thyroid cancer driven by BRAF^V600E^. Review articles discussing these models (Table 2, Fig. 3) have already been published, which include *Braf*^*V600E*^ knock-in mice crossed with cooperating alterations commonly found in advanced thyroid cancer patients whose tumors are driven by BRAF^V600E^ (e.g., TERT promoter mutations, TP53 and PI3K pathway genes alterations) (13, 14, 26, 27).

In summary, mice with thyroid-specific knock-in of *Braf*^*V600E*^ combined with cooperating genetic alterations provide a robust and clinically relevant mouse model system for studying the progression of PTC to ATC. These models, based on genomic studies of advanced thyroid cancers, closely recapitulate the genetic and phenotypic changes observed in human thyroid cancer, making them invaluable tools for understanding disease mechanisms and testing novel therapies. However, the models have limitations, such as a narrow window for testing therapies targeting ATC, as the mice deteriorate rapidly once ATCs develop (33). In addition, the tumors often contain a mixture of PTC and ATC components. Given the differential responses of PTCs and ATCs to immunotherapies and the combination of dabrafenib and trametinib, a significant challenge lies in determining whether the therapeutic response is driven by PTC, ATC or both components (39, 40, 41, 42, 43).

### Oncogenic Ras knock-in models: the foundation for Ras-driven mouse models of advanced thyroid cancers

After BRAF, activating mutations of RAS (NRAS >> HRAS > KRAS) are the next most common oncogenic drivers found in advanced thyroid cancers (8, 9, 25). The first conditional oncogenic Ras knock-in model developed was *LSL-Kras*^*G12D*^ (44). In this model, exon 1 of the mouse *Kras* gene was replaced with a floxed transcriptional termination stop element (preventing expression of mutant KRAS), followed by a mouse exon 1 genomic fragment containing the G35A mutation, which encodes KRAS^G12D^. It should be noted that the transcriptional termination stop element creates a null *Kras* allele leading to embryonic lethality in embryos homozygous for *LSL-Kras*^*G12D*^. When crossed with *TPO-Cre* to induce thyroid follicular cell expression of KRAS^G12D^, it was demonstrated that endogenous levels of oncogenic KRAS^G12D^ alone were insufficient to induce thyroid tumorigenesis (45, 46), unless the mice were made chronically hypothyroid by treatment with propylthiouracil (46), where malignant lesions developed with low penetrance and long latency (46). These results align with human studies, where fine-needle aspiration samples positive for a RAS mutation were less likely than those with *BRAF* alterations to have histologically malignant nodules (47, 48). This may be due to the fact that oncogenic RAS proteins are weaker activators of the MAPK pathway, as they are more sensitive to feedback regulation that attenuates MAPK pathway activity (49, 50, 51, 52). Recently, using CRISPR-based engineering the *Kras*^*G12D*^ in the *LSL-Kras*^*G12D*^ mice was altered to *Kras*^*G12C*^, *Kras*^*G12R*^ or *Kras*^*G13D*^ (53). In lung and pancreatic cancer models, there were striking differences between these oncogenic *Kras* variants (53). However, to date, there have been no reports utilizing these other oncogenic *Kras* variants to explore potential differences in thyroid tumorigenesis.

Mice with conditional knock-in of *Hras*^*G12V*^ (49) were generated using a flox-and-replace strategy (*FR-Hras*^*G12V*^). In this model, the coding region of the HRAS gene was replaced with a floxed wild-type mouse *Hras* gene, followed by a mouse *Hras* genomic fragment containing the G35T mutation, which encodes HRAS^G12V^. In this line, Cre recombination deletes the wild-type *Hras*, replacing it with the mutant *Hras* gene. Similar to *TPO-Cre*/*LSL-Kras*^*G12*^ mice, expression of HRAS^G12V^ alone is not sufficient to promote thyroid transformation (52, 54, 55). However, in *TPO-Cre/FR-Hras*^*G12V*^ mice that are homozygous for *Hras*^*G12V*^, thyroid hyperplasia develops after a prolonged latency (52, 54, 55).

When crossed with *p53-flox* mice, both *TPO-Cre/FR-Hras*^*G12V*^ and *TPO-Cre/LSL-Kras*^*G12D*^ models develop PDTCs and ATCs with a latency of 6–10 months, indicating that these transgenic lines provide a robust foundation for creating mouse models of oncogenic Ras-driven advanced thyroid cancers. This is further supported by studies where oncogenic Ras knock-in mice were crossed with mice harboring cooperating alterations commonly found in RAS-driven advanced thyroid cancers. These multitransgenic models that develop thyroid cancers frequently progress to PDTC and ATC. Review articles discussing these models (Table 2) have been published (13, 14, 26, 27), including examples of *Hras* or *Kras* knock-in mice crossed with cooperating genetic alterations commonly found in advanced thyroid cancers driven by oncogene RAS (e.g., NF2, EIF1AX and PI3K pathway genes).

Conditional knock-in *Nras*^*G12D*^ mice have also been developed (56); however, there are currently no reports describing thyroid-specific knock-in of *Nras*^*G12D*^. Given that NRAS is the second most common oncogenic driver in PDTCs and ATCs (8, 9, 25), developing this model is a crucial step for advancing our understanding of RAS-driven thyroid cancers.

### Role of RBM10 loss in thyroid cancer progression

*RBM10* (RNA-binding motif protein 10) is an RNA splicing factor known to influence cancer progression by regulating splicing events in specific genes. In thyroid cancer, particularly in aggressive and metastatic forms, the loss of *RBM10* has been identified as a significant factor in promoting tumor progression and metastasis. *RBM10* alterations are markedly enriched in fatal non-ATC, and in most cases, these alterations occur in combination with oncogenic Ras (57). *RBM10* mutations were also found to be associated with an increased risk of metastasis in papillary thyroid cancers (55).

To explore the role of RBM10 as a cooperating alteration in the context of oncogenic RAS drivers, Krishnamoorthy *et al.* generated mice with thyroid-specific knock-in of *Hras*^*G12V*^ and loss of *Rbm10* (*TPO-Cre/FR-Hras*^*G12V*^*/LSL-eYFP/Rbm10*^*f/f*^). After one year, none of the mice with either the *Hras*^*G12V*^ knock-in or loss of *Rbm10* alone developed thyroid neoplasia. However, most of the 10- to 12-months-old mice with both alterations developed ATC, and lung metastases were found in 18% of these mice. To identify mRNAs whose splicing is regulated by RBM10, the authors generated two isogenic human thyroid cancer cell lines with restored RBM10 expression and three isogenic human thyroid cancer cell lines with RBM10 knockdown. Among the top mRNA with RBM10-regulated exon inclusion were NUMB, SMN2, EIF4H, VCL, CD44, FN1, TPM1 and TPM3. Furthermore, using human thyroid cancer cell lines, they demonstrated that RBM10-mediated splicing of VCL, CD44 and FN1 enhanced cell migration and invasion. Using a cell line derived from a lung metastasis of a *TPO-Cre/FR-Hras*^*G12V*^*/LSL-eYFP/Rbm10*^*f/f*^ mouse, the authors showed that restoring RBM10 expression markedly reduced lung metastasis when injected into the tail vein of immunocompetent mice. The study further demonstrated that, while knockdown of the exon inclusion isoforms of VCL, CD44 or FN1 in the metastatic mouse cell line alone had no significant effect on the development of lung metastasis, the combined knockdown of all three reduced lung metastasis to levels seen after restoring RBM10. This suggests that collectively, these three exon inclusion isoforms were the key targets through which RBM10 loss promotes the metastatic spread of thyroid cancers (Fig. 3A).

In conclusion, the loss of RBM10 in cancer cells leads to extensive disruption in the splicing of mRNAs related to cytoskeletal and extracellular matrix (ECM) components. These aberrations result in structural and functional changes that significantly enhance the metastatic fitness of cancer cells. By enabling greater cell motility, promoting ECM remodeling and potentially aiding immune evasion, RBM10 loss supports cancer cell dissemination and colonization of new tissues. Targeting the mechanisms downstream of RBM10 loss, particularly the pathways governing cytoskeletal and ECM dynamics, presents a promising direction for reducing metastasis in aggressive cancers. As the complexities of RBM10’s role in metastasis are unraveled, new therapeutic opportunities are likely to emerge to help limit cancer spread and improve patient outcomes. In addition, it will be important to determine whether the tumor-specific exon inclusions mediated by RBM10 inactivation can serve as neoantigen-like targets for immunotherapies (58, 59).

### Role of miR-31 in thyroid cancer progression and dedifferentiation

MicroRNAs play a crucial role in thyroid cancer, functioning as key posttranscriptional regulators of gene expression that influence cellular differentiation, proliferation and apoptosis. Among these miRNAs, miR-31 has emerged as a significant player in thyroid cancer pathogenesis, particularly in cancers driven by the BRAF^V600E^ mutation, which is commonly associated with aggressive forms of PTC. The altered expression of miR-31 in thyroid cancer cells contributes to tumorigenesis, cancer progression and resistance to standard treatments such as radioiodine therapy, highlighting its potential as both a biomarker and a therapeutic target.

To investigate how miR-31 influences thyroid tumorigenesis and dedifferentiation process in BRAF^V600E^-driven thyroid cancer, Zhang *et al.* (60) crossed *TPO-CreER*^*T2*^ (33), *Braf*^*CA*^ (29) and *miR-31*^*f/f*^ (61) mice to generate the transgenic line *TPO-CreER*^*T2*^*/Braf*^*CA*^*/miR-31*^*f/f*^. These mice have thyroid-specific loss of miR-31 and conditional knock-in of *Braf*^*V600E*^ in thyroid follicular cells. The study demonstrated that the loss of miR-31 reduced tumor penetrance, which was nearly 100% in the *TPO-CreER*^*T2*^*/Braf*^*CA*^ mice, and the tumors that did form grew more slowly. Furthermore, it was shown that BRAF^V600E^-driven tumors lacking miR-31 maintained better differentiation and showed improved uptake of ^131^I. Using nanoparticle-mediated administration of an anti-miR-31 antagomir, the authors demonstrated that blocking miR-31 slowed tumor growth and enhanced the uptake of ^131^I in tumors from *TPO-CreER*^*T2*^*/Braf*^*CA*^ mice. The authors concluded that targeting miR-31 presents a promising therapeutic strategy to suppress tumor growth, restore cellular differentiation and resensitize cells to radioiodine therapy. Future research into miR-31 inhibitors and their combination with current therapies could potentially improve outcomes for patients with aggressive, treatment-resistant thyroid cancers.

### Role of METTL3 in thyroid cancer

Previous studies using human tissue and cell lines have suggested that the reduced expression of METTL3, an important methyltransferase responsible for N6-methyladenosine (m6A) modification of RNA, is associated with PTC progression and lymphatic metastasis (62). Ning *et al.* (63) reported that lower METTL3 levels, influenced by macrophage-derived extracellular vesicles, were associated with a reduced efficacy of anti-PD1 in both PTC and ATC patients. Decreased expression of METTL3 led to a reduction in m6A modification on specific target mRNAs, most notably CD70. Typically, m6A modification tags CD70 mRNA for degradation; thus, METTL3 inhibition allowed CD70 mRNA to accumulate, resulting in increased CD70 protein expression in thyroid cancer cells. Using xenograft mice injected with human peripheral blood mononuclear cells (PBMCs), the authors demonstrated that overexpression of METTL3 enhanced the response to anti-PD1 therapy. In contrast, METTL3 knockdown promoted resistance to anti-PD1, which could be reversed by blocking CD70 with a monoclonal antibody.

To investigate the role of *Mettl3* in a mouse model with a completely intact immune system, Kang *et al.*, (64) generated mice with a thyroid-specific knockout of *Mettl3* and a knock-in of *Braf*^*V600E*^ (*TPO-Cre/Mettl3*^*fl/fl*^*/Braf*^*CA*^). In these mice, the loss of *Mettl3* accelerated dedifferentiation and tumor progression compared to *TPO-Cre/Mettl3*^*fl/fl*^*/Braf*^*V600E*^ mice. The *Mettl3* knockout resulted in the development of larger, more aggressive tumors, increased metastatic potential (particularly pulmonary metastasis) and shorter survival times compared to *TPO-Cre/Braf*^*CA/+*^ mice. Mice with heterozygous loss of *Mettl3* displayed intermediate phenotypes, underscoring a dose-dependent effect in cooperation with BRAF^V600E^ to drive tumor progression. The body weight of *TPO-Cre/Mettl3*^*fl/fl*^*/Braf*^*CA/+*^ mice was lower than that of *TPO-Cre/Braf*^*CA/+*^ mice, suggesting more extensive thyroid dedifferentiation, although this was not directly confirmed. However, the authors’ *in vitro* studies supported this observation.

In conclusion, the study suggests that targeting METTL3 may influence the differentiation status of thyroid cancers, potentially making them more responsive to standard treatments such as chemotherapy and radioactive iodine. This could improve outcomes for patients with aggressive forms of TC, particularly those with dedifferentiated or radioiodine-refractory disease. In addition, given the increasing focus on miRNAs as therapeutic targets, inhibiting miR-493-5p could offer a strategy to elevate METTL3 levels and restore its tumor-suppressive effects. Future therapies may be able to exploit this pathway to improve outcomes in aggressive forms of thyroid cancer.

### Role of NG2 in thyroid cancer

**Figure 4:**
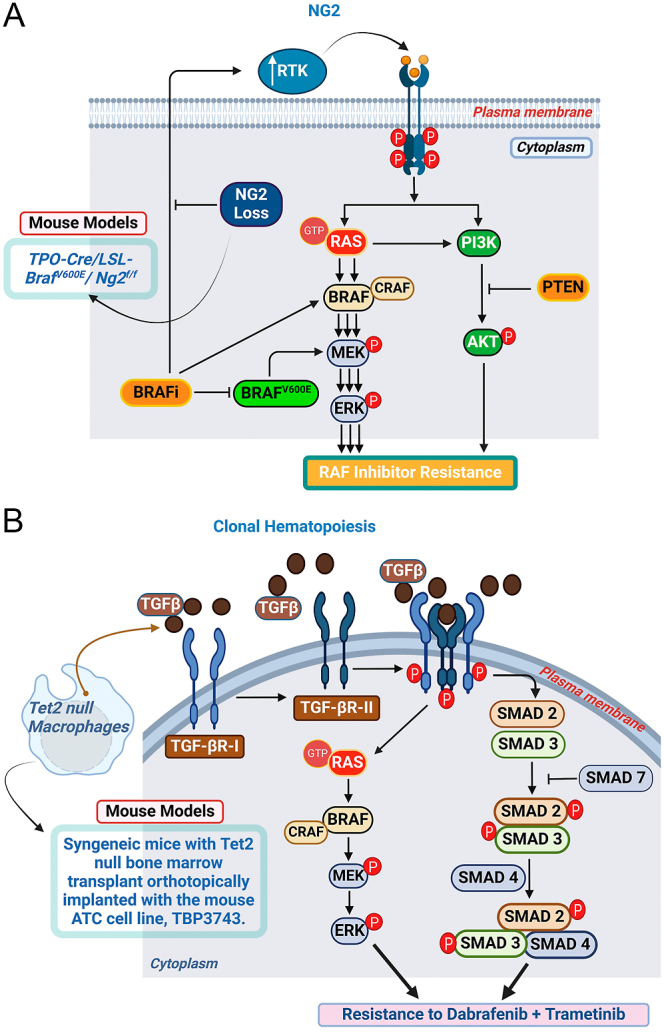
Thyroid cancer mouse models used to explore the effects of genomic alterations on the response of BRAF^V600E^-driven thyroid cancer to BRAF inhibitors. (A) NG2 overexpression. NG2 expression enhances RTK signaling, sustaining MAPK pathway activation in the presence of BRAF inhibitors and promoting resistance of BRAF^V600E^-driven thyroid cancers to these treatments. (B) CH. CH refers to somatic mutations in hematopoietic stem and progenitor cells, leading to their overrepresentation in the blood. The CH-mutant Tet2 promotes macrophage infiltration into the tumor. Once in the tumor, these macrophages secrete high levels of TGFβ, which in turn drives RAS-dependent activation of the MAPK pathway, promoting resistance to MAPK pathway inhibition by dabrafenib + trametinib. The illustration was created using BioRender.com.

NG2 (CSPG4) has been implicated in various cancers, including melanoma, glioblastoma and thyroid cancer, where its high expression correlates with poor prognosis due to its support of tumor growth, angiogenesis and resistance to therapies (65). NG2 is a cell surface proteoglycan that plays an important role in regulating RTK activity (66). In BRAF-mutant thyroid cancer, NG2 contributes to resistance against BRAF inhibitors by sustaining alternative growth signaling pathways, particularly through RTK signaling. Given the critical role of RTK activation in the adaptive resistance of BRAF^V600E^-mutant cancers to BRAF inhibitors (67, 68, 69, 70), Sui *et al.* (71) hypothesized that the overexpression of NG2, commonly observed in advanced thyroid cancers, promotes resistance to BRAF inhibitors in BRAF^V600E^-mutant thyroid cancers. To test this hypothesis, the authors generated mice with thyroid-specific knock-in of *Braf*^*V600E*^ and knockout of *Ng2* (*TPO-Cre/Ng2*^*fl/fl*^*/Braf*^*CA*^). Using this model, they demonstrated that *NG2* knockout did not significantly affect tumor growth, MAPK pathway activation, PI3K pathway activation or RTK activity under normal conditions. However, when these mice were treated with the BRAF inhibitor, PLX470, there was a significant reduction in MAPK/ERK and PI3K pathway activity in *TPO-Cre/Ng2*^*fl/fl*^*/Braf*^*CA*^ mice compared to *TPO-Cre/Braf*^*CA*^ mice. This effect was further validated by studies in thyroid cancer cell lines, which showed that the loss of NG2 reduced the BRAF inhibitor-induced feedback activation of RTKs (Fig. 4A).

In conclusion, the authors suggest that targeting NG2 represents a promising strategy to overcome resistance to BRAF inhibitors in BRAF^V600E^-driven thyroid cancer. This study underscores the importance of addressing compensatory signaling mechanisms that reactivate the MAPK pathway (68, 72), and lays the foundation for developing more effective combination therapies aimed at circumventing adaptive drug resistance.

### Mitochondrial quality control and thyroid cancer

Mitochondrial quality control is crucial for maintaining cellular homeostasis, and is primarily mediated by autophagy-related pathways and proteins. MIEAP (mitochondria-eating protein) is an autophagy-related tumor suppressor that selectively targets damaged mitochondria promoting their repair or degradation. This process helps maintain cellular homeostasis and protects cells from oxidative stress. MIEAP’s function is particularly important in cancers due to the high metabolic demands and frequent mitochondrial dysfunction observed in tumor cells. MIEAP has been shown to act as a tumor suppressor in breast, gastric and esophageal cancers by preserving mitochondrial integrity and reducing reactive oxygen species (ROS) production, which is associated with DNA damage and mutagenesis (73, 74). Another key player, ATG5 (autophagy-related gene 5), is essential for autophagosome formation, where damaged or excess cellular components are engulfed for degradation. ATG5-mediated autophagy eliminates damaged organelles and misfolded proteins, preventing cellular stress and genomic instability, both of which contribute to tumor development.

In both oncocytic and non-oncocytic thyroid cancers, MIEAP expression is often lost, likely due to epigenetic changes (75), suggesting that it may also function as a tumor suppressor in thyroid cancer. To test the hypothesis that genes involved in mitochondrial repair and degradation act as tumor suppressors, Hamada *et al.* (76) generated *Braf*^*CA*^*/Mieap*^*KO*^ and *Braf*^*CA*^*/Atg5*^*f/f*^ mice. Using the thyroid injection of Ad–TgP–Cre (77), they induced thyroid-specific knock-in of *Braf*^*V600E*^ in the context of whole-body loss of *Mieap* or knock-in of *Braf*^*V600E*^ and loss of *Atg5*. In these models, Ad–TgP–Cre-injected *Braf*^*CA*^*/Mieap*^*KO*^ and *Braf*^*CA*^*/Atg5*^*f/f*^ mice exhibited shorter latency to thyroid cancer development compared to *Braf*^*CA*^ mice. Notably, neither Ad–TgP–Cre-injected *Mieap*^*KO*^ or *Atg5*^*f/f*^ mice developed thyroid cancer within one year. Immunostaining for 53BP1, a marker of ROS-induced DNA damage, showed higher staining in tumors from *Braf*^*CA*^*/Mieap*^*KO*^ mice compared to tumors from *Braf*^*CA*^ mice, suggesting that loss of MIEAP in the context of oncogenic BRAF leads to increased ROS production.

In conclusion, the authors propose that MIEAP and ATG5 function as critical tumor suppressors in BRAF^V600E^-driven thyroid cancer by maintaining cellular integrity through distinct but complementary mechanisms. Their roles in mitochondrial quality control and autophagy highlights the importance of cellular housekeeping in countering oncogenic mutations. Future research may focus on therapeutic strategies to enhance MIEAP and ATG5 function or mimic their actions, potentially offering a novel approach for treating aggressive thyroid cancers (78, 79).

## Humanized mouse models: novel approach for investigating thyroid cancer biology

Cancer research has long relied on animal models to investigate the mechanisms underlying tumor development and test novel therapeutic approaches (reviewed in (80, 81, 82)). However, traditional mouse models of thyroid cancer often fail to fully recapitulate human cancer biology due to species-specific differences. Humanized mice bridge this gap by providing a platform where human tissues, particularly the immune system, interact with human tumors. This approach allows researchers to study tumor growth, metastasis and treatment responses in an environment more representative of human physiology. These mice are genetically engineered or grafted to possess human cells, tissues or even a human-like immune system, facilitating a more physiologically relevant model for studying human cancers. Despite their advantages, humanized mouse models also present several limitations:

i) High cost and complexity: the creation and maintenance of humanized mice are significantly more expensive and complex compared to traditional mouse models. These mice require specialized facilities, meticulous handling and additional resources, making them less accessible for many research laboratories; ii) incomplete immune system engraftment: although humanized mice aim to replicate human immune responses, engraftment is often incomplete. For instance, certain immune cell types, such as natural killer cells or myeloid cells, may not fully develop or function as they do in humans. This can limit the ability of these models to accurately predict immune responses and therapeutic outcomes, particularly for immunotherapies; iii) persistent species-specific differences: despite incorporating human components in these models, fundamental differences between mouse and human biology persist. For instance, mouse cytokine and growth factor networks can differ from their human counterparts, affecting tumor growth and immune responses. Moreover, mouse tissues may still interact differently with human cells, introducing confounding factors that could impact study results.

While humanized mice thus far have very limited use in thyroid cancer studies (63), they have the potential to significantly advance our understanding of thyroid cancer biology. These models offer a more physiologically relevant alternative to traditional mice, particularly for replicating patient-specific tumor characteristics of thyroid cancer and simulating human immune responses. As such, they could become invaluable preclinical models for studying thyroid cancer’s response to immunotherapies, cancer vaccines and targeted therapies. However, these benefits come with drawbacks, including high costs, technical challenges, incomplete immune system engraftment and lingering species-specific differences. As technology and methodologies continue to improve, future innovations are likely to address some of these limitations, making humanized mice an increasingly effective tool in the fight against thyroid cancer.

## Syngeneic mouse models of thyroid cancer

Over the past several decades, the development of genetically engineered models of advanced thyroid cancer with established phenotypes has paved the way for the development of syngeneic mouse models of thyroid cancer. One primary advantage of syngeneic models is that they maintain an intact, functional immune system, which closely replicates the tumor–immune interactions observed in human thyroid cancer. As immunotherapy becomes increasingly central to cancer treatment, syngeneic models have become invaluable for exploring immune responses and testing immunotherapeutic approaches. In thyroid cancer, the immune system plays a complex role; immune cells within the tumor microenvironment can either suppress tumor growth or contribute to tumor progression. Syngeneic models allow researchers to investigate how the immune system interacts with thyroid cancer cells across different stages of disease progression.

Because syngeneic models use immunocompetent mice, they enable the testing of checkpoint inhibitors, immune agonists and other immunomodulatory treatments within a system that closely resembles the human immune response. This offers greater translational value than immunocompromised xenograft models. Another advantage of syngeneic models is their reproducibility and cost-effectiveness compared to more complex models. Because syngeneic tumors can be generated by transplanting established cancer cell lines into mice of the same genetic background, resulting in consistent, reproducible tumor growth patterns. This consistency facilitates cross-experiment comparison. In addition, syngeneic models do not require the sophisticated breeding or maintenance associated with some genetically engineered mouse models (GEMMs), making them more accessible and cost-effective for preclinical studies. This reproducibility is crucial for studying the effects of novel drugs or immunotherapies in a controlled and repeatable manner, enabling robust preclinical testing before transitioning to more complex or costly models.

The utility of syngeneic models is exemplified in a recent study by Tiedje *et al.* (83), discussed in more detail in the next section. This study utilized a syngeneic model of ATC to investigate the effects of clonal hematopoiesis (CH) on the response to targeted therapy – a study that would have been extremely challenging, if not impossible, to conduct without a syngeneic model. However, a potential drawback of syngeneic models is that the tumors are typically fast-growing, which often does not allow sufficient time for immune editing or the development of distant metastases. In addition, not all advanced thyroid GEMMs are immediately translatable to syngeneic models due to mixed mouse backgrounds that can lead to tumor rejection. Nevertheless, several syngeneic models of thyroid cancer have been successful generated, providing essential preclinical tools for studying responses to targeted therapies and immunotherapies. These include cell lines derived from advanced thyroid cancers in *TPO-Cre/LSL-Kras*^*G12D*^*/Pten*^*f/f*^ (*84*), *TPO-CreER*^*T2*^*/Braf*^*CA*^*/Pten*^*f/f*^ (85), *TPO-CreER*^*T2*^*/Braf*^*CA*^*/Trp53*^*f/f*^ (85), *TPO-Cre/FR-Hras*^*G12V*^*/Pten*^*f/f*^ (86) and *TPO-Cre/LSL-Braf/Pten*^*f/+*^ (87) mice, which have been discussed in previously published reviews (12, 13).

## Role of CH in thyroid cancer

CH of indeterminate potential (CHIP), the most studied form of CH, arises when hematopoietic stem cells acquire mutations in genes commonly associated with myeloid malignancies, such as DNMT3A, TET2 and ASXL1. These mutations confer a growth advantage, allowing a specific clone of blood cells to expand over time. Although CHIP itself is not inherently malignant, it has been associated with increased risks of cardiovascular disease, inflammation and some cancers. In the context of solid tumors, including thyroid cancers, CH is associated with worse outcomes (88, 89, 90, 91, 92). The presence of mutated blood cell clones may modify immune responses and promote a pro-inflammatory state. However, the mechanisms by which CH contributes to a more aggressive tumor phenotype or impacts therapy response in solid tumors remain poorly understood.

To investigate the role of CH in thyroid tumorigenesis and response to therapy, Tiedje *et al.* (83) developed a mouse model mimicking CH in the context of BRAF^V600E^-driven ATC. The aim was to explore how CH influences the response to targeted therapies such as combined BRAF/MEK inhibitors. Although the combination of BRAF and MEK inhibitors is commonly used to treat BRAF^V600E^-mutant ATC patients, resistance almost always emerges, limiting their long-term effectiveness. To generate CH mice, the authors performed bone marrow transplants using either wild-type bone marrow or a 9:1 mixture of wild-type and Tet2-null bone marrow. As previously demonstrated (89), there was a marked expansion of Tet2-null leukocytes in the blood. Upon confirmation of bone marrow engraftment, the ATC cell line TBP3743 was orthotopically implanted into the mice. TBP3743 is a mouse cell line derived from ATCs generated in *TPO-CreERT/Braf*^*CA*^*/Trp53*^*f/f*^ mice (33, 85). There was no observed difference in mouse overall survival or tumor immune infiltration between mice receiving wild-type bone marrow and those receiving Tet2-null bone marrow. However, tumors in mice with Tet2-null bone marrow showed an enrichment of Tet2-null leukocytes, particularly due to the preferential infiltration of Tet2-null monocytes, dendritic cells and macrophages.

To test the hypothesis that CH alters the response to targeted therapies, the authors treated the tumor-bearing CH mice with a combination of dabrafenib and trametinib (dab/tram), a BRAF/MEK inhibitor combination commonly used for treating BRAF^V600E^-mutant ATC patients. Mice with wild-type bone marrow showed a robust anti-tumor response to dab/tram. In contrast, the response was markedly attenuated in mice with Tet2-null bone marrow, resulting in more than a two-fold decrease in overall survival compared to dab/tram-treated mice with wild-type bone marrow.

Using CITE-seq, the authors demonstrated that *Tet2*-null tumor-infiltrating macrophages produced increased levels of TGFβ, which activated the TGFβ pathway in tumor cells. This activation diminished the effectiveness of MAPK pathway inhibition by dab/tram (Fig. 4B). The role of TGFβ signaling was further confirmed by combining dab/tram with the TGFβ receptor inhibitor, vactosertib (93), or the TGFβ-depleting antibody, 1D11 (94). These combinations restored MAPK pathway inhibition, resulting in tumor regression and survival rates comparable to those in dab/tram-treated mice with wild-type bone marrow.

The findings of this study have significant implications for the clinical management of ATC, particularly in older patients who are more prone to developing CH. Since CH is associated with aging and can influence the tumor microenvironment, older patients may face a higher risk of developing treatment-resistant tumors. Testing for CH mutations such as TET2 could become a part of the diagnostic and treatment planning process for ATC, helping to identify patients who might benefit from combination therapies that include TGFβ inhibitors in combination with standard BRAF/MEK inhibitors. In addition, the study suggests a potential role for CH screening in other types of cancers, as CH-associated mutations may have broader implications for resistance mechanisms across various solid tumors.

In conclusion, mouse models of advanced thyroid cancer, created by combining oncogenic drivers with cooperating alterations that promote progression, effectively replicate many aspects of human thyroid cancers. These models provide valuable insights into the mechanisms of thyroid cancer progression, especially when informed by the genomics of human thyroid cancers. However, there is currently a lack of models that incorporate oncogenic drivers alongside alterations in histone methyltransferases (e.g., KTM2A, KTM2C and KTM2D) and mismatch repair genes (e.g., MSH2, MSH6 and MLH1), which are associated with thyroid tumor progression in humans (8). In addition, it is crucial to utilize these advanced thyroid cancer models to gain a better understanding of how various combinations of oncogenic drivers and cooperating alterations influence therapeutic responses.

## Declaration of interest

JAK is an inventor in intellectual property and holds a patent at MSKCC on a thyroid differentiation classifier for patient stratification in thyroid cancer. He helped in the treatment of H-RAS-driven tumors with FTIs and in targeting the adaptive responses of BRAF or RAS-mutant thyroid cancers to RAF/MEK inhibitors with bispecific antibodies to TSHR and/or HER2. JAK is an inventor on intellectual property and holds a patent at CCF on using fusion neoantigens as targets for cancer vaccines, antibody therapies and T-cell therapies. SD has no competing interests to declare.

## Funding

The authors are grateful for the support from DODhttps://doi.org/10.13039/100000005 grant HT94252410460 (JAK), NIHhttps://doi.org/10.13039/100000002 grants R35CA232097 (SD and JAK) and U54CA274513 (JAK).

## Author contribution statement

SD and JAK helped in writing of the original draft, reviewing and editing.

## References

[1] Hurley PM, Hill RN & Whiting RJ. Mode of carcinogenic action of pesticides inducing thyroid follicular cell tumors in rodents. Environ Health Perspect 1998 106 437–445. (10.2307/3434175) 9681970 PMC1533205

[2] Knostman KA, Jhiang SM & Capen CC. Genetic alterations in thyroid cancer: the role of mouse models. Vet Pathol 2007 44 1–14. (10.1354/vp.44-1-1) 17197619

[3] Kim CS & Zhu X. Lessons from mouse models of thyroid cancer. Thyroid 2009 19 1317–1331. (10.1089/thy.2009.1609) 20001715 PMC2861953

[4] Zhu XG & Cheng SY. Modeling thyroid cancer in the mouse. Horm Metab Res 2009 41 488–499. (10.1055/s-0029-1215572) 19358084 PMC3464089

[5] Rusinek D, Krajewska J & Jarzab M. Mouse models of papillary thyroid carcinoma – short review. Endokrynol Pol 2016 67 212–223. (10.5603/EP.a2016.0042) 27082155

[6] Cancer Genome Atlas Research Network. Integrated genomic characterization of papillary thyroid carcinoma. Cell 2014 159 676–690. (10.1016/j.cell.2014.09.050) 25417114 PMC4243044

[7] Kunstman JW, Juhlin CC, Goh G, et al. Characterization of the mutational landscape of anaplastic thyroid cancer via whole-exome sequencing. Hum Mol Genet 2015 24 2318–2329. (10.1093/hmg/ddu749) 25576899 PMC4380073

[8] Landa I, Ibrahimpasic T, Boucai L, et al. Genomic and transcriptomic hallmarks of poorly differentiated and anaplastic thyroid cancers. J Clin Investig 2016 126 1052–1066. (10.1172/jci85271) 26878173 PMC4767360

[9] Pozdeyev N, Gay LM, Sokol ES, et al. Genetic analysis of 779 advanced differentiated and anaplastic thyroid cancers. Clin Cancer Res 2018 24 3059–3068. (10.1158/1078-0432.ccr-18-0373) 29615459 PMC6030480

[10] Charles RP. Overview of genetically engineered mouse models of papillary and anaplastic thyroid cancers: enabling translational biology for patient care improvement. Curr Protoc Pharmacol 2015 69 1–14. (10.1002/0471141755.ph1433s69) 26344211

[11] Jin Y, Liu M, Sa R, et al. Mouse models of thyroid cancer: bridging pathogenesis and novel therapeutics. Cancer Lett 2020 469 35–53. (10.1016/j.canlet.2019.09.017) 31589905

[12] Jeon MJ & Haugen BR. Preclinical models of follicular cell-derived thyroid cancer: an overview from cancer cell lines to mouse models. Endocrinol Metab 2022 37 830–838. (10.3803/enm.2022.1636) PMC9816502 36604954

[13] Choi HR & Kim K. Mouse models to examine differentiated thyroid cancer pathogenesis: recent updates. Int J Mol Sci 2023 24 11138. (10.3390/ijms241311138) 37446316 PMC10342769

[14] Xu B & Ghossein R. Genomic landscape of poorly differentiated and anaplastic thyroid carcinoma. Endocr Pathol 2016 27 205–212. (10.1007/s12022-016-9445-4) 27372303

[15] Yoo SK, Song YS, Park YJ, et al. Recent improvements in genomic and transcriptomic understanding of anaplastic and poorly differentiated thyroid cancers. Endocrinol Metab 2020 35 44–54. (10.3803/enm.2020.35.1.44) PMC7090308 32207263

[16] Abe I & Lam AK. Anaplastic thyroid carcinoma: current issues in genomics and therapeutics. Curr Oncol Rep 2021 23 31. (10.1007/s11912-021-01019-9) 33582932

[17] Macerola E, Poma AM, Vignali P, et al. Molecular genetics of follicular-derived thyroid cancer. Cancers 2021 13 1139. (10.3390/cancers13051139) 33799953 PMC7961716

[18] Singh A, Ham J, Po JW, et al. The genomic landscape of thyroid cancer tumourigenesis and implications for immunotherapy. Cells 2021 10 1082. (10.3390/cells10051082) 34062862 PMC8147376

[19] Fagin JA, Krishnamoorthy GP & Landa I. Pathogenesis of cancers derived from thyroid follicular cells. Nat Rev Cancer 2023 23 631–650. (10.1038/s41568-023-00598-y) 37438605 PMC10763075

[20] Fagin JA & Nikiforov YE. Progress in thyroid cancer genomics: a 40-year journey. Thyroid 2023 33 1271–1286. (10.1089/thy.2023.0045) 37668657 PMC10664575

[21] Leandro-Garcia LJ & Landa I. Mechanistic insights of thyroid cancer progression. Endocrinology 2023 164 bqad118. (10.1210/endocr/bqad118) 37503738 PMC10403681

[22] Khan SA, Ci B, Xie Y, et al. Unique mutation patterns in anaplastic thyroid cancer identified by comprehensive genomic profiling. Head Neck 2019 41 1928–1934. (10.1002/hed.25634) 30758123 PMC6542589

[23] Ravi N, Yang M, Gretarsson S, et al. Identification of targetable lesions in anaplastic thyroid cancer by genome profiling. Cancers 2019 11 402. (10.3390/cancers11030402) 30909364 PMC6468430

[24] Yoo SK, Song YS, Lee EK, et al. Integrative analysis of genomic and transcriptomic characteristics associated with progression of aggressive thyroid cancer. Nat Commun 2019 10 2764. (10.1038/s41467-019-10680-5) 31235699 PMC6591357

[25] Saito Y, Kage H, Kobayashi K, et al. Comprehensive genomic profiling from C-CAT database unveiled over 80% presence of oncogenic drivers in anaplastic thyroid carcinoma including BRAF, RAS family, NF1, and FGFR1. Clin Endocrinol 2024 101 170–179. (10.1111/cen.15098) 38853441

[26] Champa D & Di Cristofano A. Modeling anaplastic thyroid carcinoma in the mouse. Horm Cancer 2015 6 37–44. (10.1007/s12672-014-0208-8) 25420535 PMC4312228

[27] Landa I & Knauf JA. Mouse models as a tool for understanding progression in Braf(V600E)-driven thyroid cancers. Endocrinol Metab 2019 34 11–22. (10.3803/enm.2019.34.1.11) PMC6435851 30784243

[28] Mercer K, Giblett S, Green S, et al. Expression of endogenous oncogenic V600EB-raf induces proliferation and developmental defects in mice and transformation of primary fibroblasts. Cancer Res 2005 65 11493–11500. (10.1158/0008-5472.can-05-2211) 16357158 PMC2640458

[29] Dankort D, Filenova E, Collado M, et al. A new mouse model to explore the initiation, progression, and therapy of ^V600E^-induced lung tumors. Genes Dev 2007 21 379–384. (10.1101/gad.1516407) 17299132 PMC1804325

[30] Franco AT, Malaguarnera R, Refetoff S, et al. Thyrotrophin receptor signaling dependence of Braf-induced thyroid tumor initiation in mice. Proc Natl Acad Sci U S A 2011 108 1615–1620. (10.1073/pnas.1015557108) 21220306 PMC3029699

[31] Kusakabe T, Kawaguchi A, Kawaguchi R, et al. Thyrocyte-specific expression of Cre recombinase in transgenic mice. Genesis 2004 39 212–216. (10.1002/gene.20043) 15282748

[32] Zhang P, Guan H, Yuan S, et al. Targeting myeloid derived suppressor cells reverts immune suppression and sensitizes BRAF-mutant papillary thyroid cancer to MAPK inhibitors. Nat Commun 2022 13 1588. (10.1038/s41467-022-29000-5) 35332119 PMC8948260

[33] McFadden DG, Vernon A, Santiago PM, et al. p53 constrains progression to anaplastic thyroid carcinoma in a Braf-mutant mouse model of papillary thyroid cancer. Proc Natl Acad Sci U S A 2014 111 E1600–E1609. (10.1073/pnas.1404357111) 24711431 PMC4000830

[34] Zou M, Baitei EY, Al-Rijjal RA, et al. TSH overcomes Braf(V600E)-induced senescence to promote tumor progression via downregulation of p53 expression in papillary thyroid cancer. Oncogene 2016 35 1909–1918. (10.1038/onc.2015.253) 26477313 PMC6310059

[35] Yan H, Ma Y, Zhou X, et al. Spontaneous murine model of anaplastic thyroid cancer. J Vis Exp 2023 192 e64607. (10.3791/64607) 36804915

[36] Ryder M, Ghossein RA, Ricarte-Filho JC, et al. Increased density of tumor-associated macrophages is associated with decreased survival in advanced thyroid cancer. Endocr Relat Cancer 2008 15 1069–1074. (10.1677/erc-08-0036) 18719091 PMC2648614

[37] Giannini R, Moretti S, Ugolini C, et al. Immune profiling of thyroid carcinomas suggests the existence of two major phenotypes: an ATC-like and a PDTC-like. J Clin Endocrinol Metab 2019 104 3557–3575. (10.1210/jc.2018-01167) 30882858

[38] Han PZ, Ye WD, Yu PC, et al. A distinct tumor microenvironment makes anaplastic thyroid cancer more lethal but immunotherapy sensitive than papillary thyroid cancer. JCI Insight 2024 9 e173712. (10.1172/jci.insight.173712) 38478516 PMC11141884

[39] Shah MH, Wei L, Wirth LJ, et al. Results of randomized phase II trial of dabrafenib versus dabrafenib plus trametinib in BRAF-mutated papillary thyroid carcinoma. Am Soc Clin Oncol 2017 35 6022. (10.1200/jco.2017.35.15_suppl.6022)

[40] Capdevila J, Wirth LJ, Ernst T, et al. PD-1 blockade in anaplastic thyroid carcinoma. J Clin Oncol 2020 38 2620–2627. (10.1200/jco.19.02727) 32364844 PMC7476256

[41] Busaidy NL, Konda B, Wei L, et al. Dabrafenib versus dabrafenib + trametinib in BRAF-mutated radioactive iodine refractory differentiated thyroid cancer: results of a randomized, phase 2, open-label multicenter trial. Thyroid 2022 32 1184–1192. (10.1089/thy.2022.0115) 35658604 PMC9595631

[42] Subbiah V, Kreitman RJ, Wainberg ZA, et al. Dabrafenib plus trametinib in patients with BRAF V600E-mutant anaplastic thyroid cancer: updated analysis from the phase II ROAR basket study. Ann Oncol 2022 33 406–415. (10.1016/j.annonc.2021.12.014) 35026411 PMC9338780

[43] Oh DY, Algazi A, Capdevila J, et al. Efficacy and safety of pembrolizumab monotherapy in patients with advanced thyroid cancer in the phase 2 KEYNOTE-158 study. Cancer 2023 129 1195–1204. (10.1002/cncr.34657) 36748723

[44] Jackson EL, Willis N, Mercer K, et al. Analysis of lung tumor initiation and progression using conditional expression of oncogenic K-ras. Genes Dev 2001 15 3243–3248. (10.1101/gad.943001) 11751630 PMC312845

[45] Champa D, Russo MA, Liao XH, et al. Obatoclax overcomes resistance to cell death in aggressive thyroid carcinomas by countering Bcl2a1 and Mcl1 overexpression. Endocr Relat Cancer 2014 21 755–767. (10.1530/erc-14-0268) 25012986 PMC4152557

[46] Zou M, Baitei EY, Al-Rijjal RA, et al. KRAS-mediated oncogenic transformation of thyroid follicular cells requires long-term TSH stimulation and is regulated by SPRY1. Lab Invest 2015 95 1269–1277. (10.1038/labinvest.2015.90) 26146959 PMC6289253

[47] Skaugen JM, Taneja C, Liu JB, et al. Performance of a multigene genomic classifier in thyroid nodules with suspicious for malignancy cytology. Thyroid 2022 32 1500–1508. (10.1089/thy.2022.0282) 35864811 PMC9807251

[48] Yang SP, Nga ME, Bundele MM, et al. Performance of a multigene genomic classifier and clinical parameters in predicting malignancy in a Southeast Asian cohort of patients with cytologically indeterminate thyroid nodules. Cancer Cytopathol 2024 132 309–319. (10.1002/cncy.22796) 38319805

[49] Chen X, Mitsutake N, LaPerle K, et al. Endogenous expression of Hras(G12V) induces developmental defects and neoplasms with copy number imbalances of the oncogene. Proc Natl Acad Sci U S A 2009 106 7979–7984. (10.1073/pnas.0900343106) 19416908 PMC2674938

[50] Miller KA, Yeager N, Baker K, et al. Oncogenic Kras requires simultaneous PI3K signaling to induce ERK activation and transform thyroid epithelial cells in vivo. Cancer Res 2009 69 3689–3694. (10.1158/0008-5472.can-09-0024) 19351816 PMC2669852

[51] Chen X, Makarewicz JM, Knauf JA, et al. Transformation by Hras(G12V) is consistently associated with mutant allele copy gains and is reversed by farnesyl transferase inhibition. Oncogene 2014 33 5442–5449. (10.1038/onc.2013.489) 24240680 PMC4025988

[52] Garcia-Rendueles ME, Ricarte-Filho JC, Untch BR, et al. NF2 loss promotes oncogenic RAS-induced thyroid cancers via YAP-dependent transactivation of RAS proteins and sensitizes them to MEK inhibition. Cancer Discov 2015 5 1178–1193. (10.1158/2159-8290.cd-15-0330) 26359368 PMC4642441

[53] Zafra MP, Parsons MJ, Kim J, et al. An in vivo Kras allelic series reveals distinct phenotypes of common oncogenic variants. Cancer Discov 2020 10 1654–1671. (10.1158/2159-8290.cd-20-0442) 32792368 PMC7642097

[54] Montero-Conde C, Leandro-Garcia LJ, Chen X, et al. Transposon mutagenesis identifies chromatin modifiers cooperating with ras in thyroid tumorigenesis and detects ATXN7 as a cancer gene. Proc Natl Acad Sci U S A 2017 114 E4951–E4960. (10.1073/pnas.1702723114) 28584132 PMC5488945

[55] Krishnamoorthy GP, Glover AR, Untch BR, et al. RBM10 loss induces aberrant splicing of cytoskeletal and extracellular matrix mRNAs and promotes metastatic fitness. bioRxiv 2024. (10.1101/2024.07.09.602730) PMC11849553 39992626

[56] Haigis KM, Kendall KR, Wang Y, et al. Differential effects of oncogenic K-ras and N-ras on proliferation, differentiation and tumor progression in the colon. Nat Genet 2008 40 600–608. (10.1038/ng.115) 18372904 PMC2410301

[57] Ibrahimpasic T, Xu B, Landa I, et al. Genomic alterations in fatal forms of non-anaplastic thyroid cancer: identification of MED12 and RBM10 as novel thyroid cancer genes associated with tumor virulence. Clin Cancer Res 2017 23 5970–5980. (10.1158/1078-0432.ccr-17-1183) 28634282 PMC5626586

[58] Bernard A, Boidot R & Vegran F. Alternative splicing in cancer and immune cells. Cancers 2022 14 1726. (10.3390/cancers14071726) 35406498 PMC8996879

[59] Huang P, Wen F, Tuerhong N, et al. Neoantigens in cancer immunotherapy: focusing on alternative splicing. Front Immunol 2024 15 1437774. (10.3389/fimmu.2024.1437774) 39055714 PMC11269099

[60] Zhang P, Guan L, Sun W, et al. Targeting miR-31 represses tumourigenesis and dedifferentiation of BRAF(V600E)-associated thyroid carcinoma. Clin Transl Med 2024 14 e1694. (10.1002/ctm2.1694) 38797942 PMC11128713

[61] Lv C, Li F, Li X, et al. MiR-31 promotes mammary stem cell expansion and breast tumorigenesis by suppressing Wnt signaling antagonists. Nat Commun 2017 8 1036. (10.1038/s41467-017-01059-5) 29051494 PMC5648844

[62] Zhou X, Chang L, Liang Q, et al. The m6A methyltransferase METTL3 drives thyroid cancer progression and lymph node metastasis by targeting LINC00894. Cancer Cell Int 2024 24 47. (10.1186/s12935-024-03240-5) 38291427 PMC10826051

[63] Ning J, Hou X, Hao J, et al. METTL3 inhibition induced by M2 macrophage-derived extracellular vesicles drives anti-PD-1 therapy resistance via M6A-CD70-mediated immune suppression in thyroid cancer. Cell Death Differ 2023 30 2265–2279. (10.1038/s41418-023-01217-x) 37648786 PMC10589295

[64] Kang N, Zhao Z, Wang Z, et al. METTL3 regulates thyroid cancer differentiation and chemosensitivity by modulating PAX8. Int J Biol Sci 2024 20 3426–3441. (10.7150/ijbs.84797) 38993572 PMC11234206

[65] Price MA, Colvin Wanshura LE, Yang J, et al. CSPG4, a potential therapeutic target, facilitates malignant progression of melanoma. Pigment Cell Melanoma Res 2011 24 1148–1157. (10.1111/j.1755-148x.2011.00929.x) 22004131 PMC3426219

[66] Lanzi C & Cassinelli G. Receptor tyrosine kinases and heparan sulfate proteoglycans: interplay providing anticancer targeting strategies and new therapeutic opportunities. Biochem Pharmacol 2020 178 114084. (10.1016/j.bcp.2020.114084) 32526230

[67] Lito P, Pratilas CA, Joseph EW, et al. Relief of profound feedback inhibition of mitogenic signaling by RAF inhibitors attenuates their activity in BRAFV600E melanomas. Cancer Cell 2012 22 668–682. (10.1016/j.ccr.2012.10.009) 23153539 PMC3713778

[68] Montero-Conde C, Ruiz-Llorente S, Dominguez JM, et al. Relief of feedback inhibition of HER3 transcription by RAF and MEK inhibitors attenuates their antitumor effects in BRAF-mutant thyroid carcinomas. Cancer Discov 2013 3 520–533. (10.1158/2159-8290.cd-12-0531) 23365119 PMC3651738

[69] Cheng L, Jin Y, Liu M, et al. HER inhibitor promotes BRAF/MEK inhibitor-induced redifferentiation in papillary thyroid cancer harboring BRAFV600E. Oncotarget 2017 8 19843–19854. (10.18632/oncotarget.15773) 28423638 PMC5386727

[70] Dang H, Sui M, He Q, et al. Pin1 inhibitor API-1 sensitizes BRAF-mutant thyroid cancers to BRAF inhibitors by attenuating HER3-mediated feedback activation of MAPK/ERK and PI3K/AKT pathways. Int J Biol Macromol 2023 248 125867. (10.1016/j.ijbiomac.2023.125867) 37473892

[71] Sui F, Wang G, Liu J, et al. Targeting NG2 relieves the resistance of BRAF-mutant thyroid cancer cells to BRAF inhibitors. Cell Mol Life Sci 2024 81 238. (10.1007/s00018-024-05280-6) 38795180 PMC11127897

[72] Garcia-Rendueles MER, Krishnamoorthy G, Saqcena M, et al. Yap governs a lineage-specific neuregulin1 pathway-driven adaptive resistance to RAF kinase inhibitors. Mol Cancer 2022 21 213. (10.1186/s12943-022-01676-9) 36476495 PMC9730579

[73] Sano H, Futamura M, Gaowa S, et al. p53/Mieap-regulated mitochondrial quality control plays an important role as a tumor suppressor in gastric and esophageal cancers. Biochem Biophys Res Commun 2020 529 582–589. (10.1016/j.bbrc.2020.05.168) 32736677

[74] Futamura M, Tokumaru Y, Takabe K, et al. MIEAP, a p53-downstream gene, is associated with suppression of breast cancer cell proliferation and better survival. Am J Cancer Res 2021 11 6060–6073. 35018242 PMC8727819

[75] Mussazhanova Z, Shimamura M, Kurashige T, et al. Causative role for defective expression of mitochondria-eating protein in accumulation of mitochondria in thyroid oncocytic cell tumors. Cancer Sci 2020 111 2814–2823. (10.1111/cas.14501) 32458504 PMC7419045

[76] Hamada K, Kurashige T, Shimamura M, et al. MIEAP and ATG5 are tumor suppressors in a mouse model of BRAF(V600E)-positive thyroid cancer. Front Endocrinol 2022 13 932754. (10.3389/fendo.2022.932754) PMC9519861 36187114

[77] Shimamura M, Nakahara M, Orim F, et al. Postnatal expression of BRAFV600E does not induce thyroid cancer in mouse models of thyroid papillary carcinoma. Endocrinology 2013 154 4423–4430. (10.1210/en.2013-1174) 23970782

[78] Zacharioudakis E & Gavathiotis E. Mitochondrial dynamics proteins as emerging drug targets. Trends Pharmacol Sci 2023 44 112–127. (10.1016/j.tips.2022.11.004) 36496299 PMC9868082

[79] Gao Y & Zheng H. Role of mitochondria and potential of mitochondria-targeted therapy in BRAF mutant cancer: a review. Crit Rev Oncol Hematol 2024 203 104484. (10.1016/j.critrevonc.2024.104484) 39197669

[80] Chen A, Neuwirth I & Herndler-Brandstetter D. Modeling the tumor microenvironment and cancer immunotherapy in next-generation humanized mice. Cancers 2023 15 2989. (10.3390/cancers15112989) 37296949 PMC10251926

[81] Chuprin J, Buettner H, Seedhom MO, et al. Humanized mouse models for immuno-oncology research. Nat Rev Clin Oncol 2023 20 192–206. (10.1038/s41571-022-00721-2) 36635480 PMC10593256

[82] Kaushik S, Kumari L & Deepak RK. Humanized mouse model for vaccine evaluation: an overview. Clin Exp Vaccin Res 2024 13 10–20. (10.7774/cevr.2024.13.1.10) PMC10864885 38362371

[83] Tiedje V, Vela PS, Yang JL, et al. Targetable treatment resistance in thyroid cancer with clonal hematopoiesis. bioRxiv 2024. (10.1101/2024.10.10.617685)

[84] Dima M, Miller KA, Antico-Arciuch VG, et al. Establishment and characterization of cell lines from a novel mouse model of poorly differentiated thyroid carcinoma: powerful tools for basic and preclinical research. Thyroid 2011 21 1001–1007. (10.1089/thy.2011.0030) 21767142 PMC3162646

[85] Vanden Borre P, McFadden DG, Gunda V, et al. The next generation of orthotopic thyroid cancer models: immunocompetent orthotopic mouse models of BRAF V600E-positive papillary and anaplastic thyroid carcinoma. Thyroid 2014 24 705–714. (10.1089/thy.2013.0483) 24295207 PMC3993062

[86] Caperton CO, Jolly LA, Massoll N, et al. Development of novel follicular thyroid cancer models which progress to poorly differentiated and anaplastic thyroid cancer. Cancers 2021 13 1094. (10.3390/cancers13051094) 33806425 PMC7961488

[87] Branigan GP, Casado-Medrano V, O'Neill AB, et al. Development of novel murine BRAF(V600E)-driven papillary thyroid cancer cell lines for modeling of disease progression and preclinical evaluation of therapeutics. Cancers 2023 15 879. (10.3390/cancers15030879) 36765847 PMC9913801

[88] Coombs CC, Zehir A, Devlin SM, et al. Therapy-related clonal hematopoiesis in patients with non-hematologic cancers is common and associated with adverse clinical outcomes. Cell Stem Cell 2017 21 374–382.e4. (10.1016/j.stem.2017.07.010) 28803919 PMC5591073

[89] Fuster JJ, MacLauchlan S, Zuriaga MA, et al. Clonal hematopoiesis associated with TET2 deficiency accelerates atherosclerosis development in mice. Science 2017 355 842–847. (10.1126/science.aag1381) 28104796 PMC5542057

[90] Boucai L, Falcone J, Ukena J, et al. Radioactive iodine-related clonal hematopoiesis in thyroid cancer is common and associated with decreased survival. J Clin Endocrinol Metab 2018 103 4216–4223. (10.1210/jc.2018-00803) 30137527 PMC6194804

[91] Bolton KL, Ptashkin RN, Gao T, et al. Cancer therapy shapes the fitness landscape of clonal hematopoiesis. Nat Genet 2020 52 1219–1226. (10.1038/s41588-020-00710-0) 33106634 PMC7891089

[92] Sanchez Vela P, Trowbridge JJ & Levine RL. Clonal hematopoiesis, aging and Alzheimer's disease. Nat Med 2023 29 1605–1606. (10.1038/s41591-023-02406-4) 37402877

[93] Jin CH, Krishnaiah M, Sreenu D, et al. Discovery of *N*-((4-([1,2,4]triazolo[1,5-*a*]pyridin-6-yl)-5-(6-methylpyridin-2-yl)-1*H*-imidazole-2-yl)methyl)-2-fluoroaniline (EW-7197): a highly potent, selective, and orally bioavailable inhibitor of TGF-β type I receptor kinase as cancer immunotherapeutic/antifibrotic agent. J Med Chem 2014 57 4213–4238. (10.1021/jm500115w) 24786585

[94] Dasch JR, Pace DR, Waegell W, et al. Monoclonal antibodies recognizing transforming growth factor-beta. Bioactivity neutralization and transforming growth factor beta 2 affinity purification. J Immunol 1989 142 1536–1541. (10.4049/jimmunol.142.5.1536) 2537357

[95] Jhiang SM, Sagartz JE, Tong Q, et al. Targeted expression of the ret/PTC1 oncogene induces papillary thyroid carcinomas. Endocrinology 1996 137 375–378. (10.1210/endo.137.1.8536638) 8536638

[96] Santoro M, Chiappetta G, Cerrato A, et al. Development of thyroid papillary carcinomas secondary to tissue-specific expression of the RET/PTC1 oncogene in transgenic mice. Oncogene 1996 12 1821–1826. 8622903

[97] Rochefort P, Caillou B, Michiels FM, et al. Thyroid pathologies in transgenic mice expressing a human activated ras gene driven by a thyroglobulin promoter. Oncogene. 1996 12 111–118. 8552381

[98] Powell DJ Jr, Russell J, Nibu K, et al. The RET/PTC3 oncogene: metastatic solid-type papillary carcinomas in murine thyroids. Cancer Res 1998 58 5523–5528. 9850089

[99] Russell JP, Powell DJ, Cunnane M, et al. The TRK-T1 fusion protein induces neoplastic transformation of thyroid epithelium. Oncogene 2000 19 5729–5735. (10.1038/sj.onc.1203922) 11126359

[100] Knauf JA, Ma X, Smith EP, et al. Targeted expression of BRAFV600E in thyroid cells of transgenic mice results in papillary thyroid cancers that undergo dedifferentiation. Cancer Res 2005 65 4238–4245. (10.1158/0008-5472.can-05-0047) 15899815

[101] Knauf JA, Sartor MA, Medvedovic M, et al. Progression of BRAF-induced thyroid cancer is associated with epithelial–mesenchymal transition requiring concomitant MAP kinase and TGFβ signaling. Oncogene 2011 30 3153–3162. (10.1038/onc.2011.44) 21383698 PMC3136543

[102] Vitagliano D, Portella G, Troncone G, et al. Thyroid targeting of the N-ras(Gln61Lys) oncogene in transgenic mice results in follicular tumors that progress to poorly differentiated carcinomas. Oncogene 2006 25 5467–5474. (10.1038/sj.onc.1209527) 16785999

[103] Charles RP, Iezza G, Amendola E, et al. Mutationally activated BRAF(V600E) elicits papillary thyroid cancer in the adult mouse. Cancer Res 2011 71 3863–3871. (10.1158/0008-5472.can-10-4463) 21512141 PMC3107361

[104] Chakravarty D, Santos E, Ryder M, et al. Small-molecule MAPK inhibitors restore radioiodine incorporation in mouse thyroid cancers with conditional BRAF activation. J Clin Investig 2011 121 4700–4711. (10.1172/jci46382) 22105174 PMC3225989

[105] Nikitski AV, Rominski SL, Wankhede M, et al. Mouse model of poorly differentiated thyroid carcinoma driven by STRN-ALK fusion. Am J Pathol 2018 188 2653–2661. (10.1016/j.ajpath.2018.07.012) 30125543 PMC6222272

[106] Schoultz E, Liang S, Carlsson T, et al. Tissue specificity of oncogenic BRAF targeted to lung and thyroid through a shared lineage factor. iScience 2023 26 107071. (10.1016/j.isci.2023.107071) 37534159 PMC10391731

[107] La Perle KM, Jhiang SM & Capen CC. Loss of p53 promotes anaplasia and local invasion in ret/PTC1-induced thyroid carcinomas. Am J Pathol 2000 157 671–677. (10.1016/s0002-9440(10)64577-4) 10934169 PMC1850128

[108] Powell DJ Jr, Russell JP, Li G, et al. Altered gene expression in immunogenic poorly differentiated thyroid carcinomas from RET/PTC3p53-/- mice. Oncogene 2001 20 3235–3246. (10.1038/sj.onc.1204425) 11423973

[109] Charles RP, Silva J, Iezza G, et al. Activating BRAF and PIK3CA mutations cooperate to promote anaplastic thyroid carcinogenesis. Mol Cancer Res 2014 12 979–986. (10.1158/1541-7786.mcr-14-0158-t) 24770869 PMC4635659

[110] Jolly LA, Massoll N & Franco AT. Immune suppression mediated by myeloid and lymphoid derived immune cells in the tumor microenvironment facilitates progression of thyroid cancers driven by Hras(G12V) and pten loss. J Clin Cell Immunol 2016 7 451. (10.4172/2155-9899.1000451) 27942419 PMC5145275

[111] Knauf JA, Luckett KA, Chen KY, et al. Hgf/Met activation mediates resistance to BRAF inhibition in murine anaplastic thyroid cancers. J Clin Investig 2018 128 4086–4097. (10.1172/jci120966) 29990309 PMC6118575

[112] Untch BR, Dos Anjos V, Garcia-Rendueles MER, et al. Tipifarnib inhibits HRAS-driven dedifferentiated thyroid cancers. Cancer Res 2018 78 4642–4657. (10.1158/0008-5472.can-17-1925) 29760048 PMC6095730

[113] Nikitski AV, Rominski SL, Condello V, et al. Mouse model of thyroid cancer progression and dedifferentiation driven by STRN-ALK expression and loss of p53: evidence for the existence of two types of poorly differentiated carcinoma. Thyroid 2019 29 1425–1437. (10.1089/thy.2019.0284) 31298630 PMC6797076

[114] Saqcena M, Leandro-Garcia LJ, Maag JLV, et al. SWI/SNF complex mutations promote thyroid tumor progression and insensitivity to redifferentiation therapies. Cancer Discov 2021 11 1158–1175. (10.1158/2159-8290.cd-20-0735) 33318036 PMC8102308

[115] Landa I, Thornton CE, Xu B, et al. Telomerase upregulation induces progression of mouse Braf^V600E^-driven thyroid cancers and triggers non-telomeric effects. Mol Cancer Res 2023 21 1163–1175. (10.1158/1541-7786.MCR-23-0144) 37478162 PMC11193891

